# Bioactivity of *Amaranthus spinosus* L. leaf extracts and meals against *Aeromonas hydrophila*

**DOI:** 10.1099/acmi.0.000305

**Published:** 2022-02-03

**Authors:** Jenny Marie Remonde Patalinghug, Mary Hannah Rose Abadinas Padayao, Isagani Pablo Angeles, Jonie Calisogan Yee

**Affiliations:** ^1^​ Department of Biology, University of San Carlos, Cebu City 6000, Philippines; ^2^​ Freshwater Fisheries Center of Cagayan Valley, Isabela State University, Echague, Philippines

**Keywords:** *Amaranthus spinosus*, plant-based, *Aeromonas hydrophila*, challenge test, Nile Tilapia, fish meals

## Abstract

Plant-based protein is being sought after as a substitute for fish meals (powdered fish) in tilapia feeds. This is to promote sustainable aquaculture, as fish meals contribute to the dwindling marine fish catch. *Amaranthus spinosus* is an edible weed that shows potential to improve the growth and immunity of Nile tilapia. However, most studies only consider the survivability of fish to evaluate the benefit of using plant-based feeds and do not necessarily elucidate whether a pathogen is affected *in vivo*. *A. spinosus* leaf meals (ASLMs) were used to determine effectiveness against *Aeromonas hydrophila* (BIOTECH 10089) injected intraperitoneally into Nile tilapia. Formulated feeds with fish meals substituted with 50 % (ASLM_50_) and 75 % (ASLM_75_) *A. spinosus* leaves were fed to Nile tilapia challenged with *A. hydrophila *. Then spleen and kidney tissue were collected and analysed 10 days post-injection for total plate count. The fish fed with ASLM_50_ appeared healthier than those fed with ASLM_75_ and those fed with control feeds. Fish fed with ASLMs had lower *A. hydrophila* counts (*P*=0.03). Phytochemical screening and antimicrobial activity determination for crude methanolic *A. spinosus* leaf (ASL) and ASLMs were also conducted to enhance the *in vivo* results. The metabolites present in the extracts were carbohydrates, amino acids and proteins, cardiac glycosides, saponins and terpenoids. The ASL and ASLM extracts had antimicrobial activity (MIC=115 mg ml^−1^). Overall, the study showed that ASLMs can make tilapia more resilient against *A. hydrophila* infections. Fish meal substitution was best at 50 %. Higher substitution had unwanted effects (more bacterial counts), possibly due to antinutritional factors.

## Impact Statement

This paper demonstrates how the leaves of an invasive plant species (*Amaranthus spinosus*) were used as a substitute for fish meals in Nile tilapia feeds. It provides evidence that the plant stimulates fish resilience against infections by *Aeromonas hydrophila *. This shows that it has beneficial metabolites that may reduce the use of synthetic antibiotics in fish aquaculture. The study promotes the development of sustainable feeds for the aquaculture industry.

## Introduction

Tilapia is a freshwater cichlid fish and one of the most farmed fishes; ~40 % of fish in aquaculture are derived from it [1]. Tilapia aquaculture is important; however, farming tilapia has been difficult because of the high cost of commercial feed. Fish meals comprise the protein component found in most aquaculture feeds and the use of fish meals has proven to be unsustainable [2]. They compete with the fish supply meant for human consumption and, sadly, global fish catch has been decreasing over the years. Another problem for tilapia farmers is low yield caused by bacterial infections such as *Aeromonas hydrophila *, which is a Gram-negative, rod-shaped bacterium, and can thrive in fresh and brackish water. It is also known to have a worldwide distribution and to be a major fish pathogen [3]. It can cause haemorrhagic septicaemia, tail/fin rot and ulcers [3]. Antibiotic resistance of *A. hydrophila* in fish has already been documented [4].

*Amaranthus spinosus* L., or ‘pigweed’, is an annual edible plant known to possess medicinal properties, including being an antimicrobial agent [5]. Its leaves are known to have high protein content [6], making it a possible substitute for fish meals. Given this, *A. spinosus* L. would not only be a sustainable alternative to fish meals, but can also make fish more resilient against bacterial infections. A study showed that Nile tilapia (*Oreochromis niloticus*) fed with feeds infused with *A. spinosus* leaves had better growth, survivability and immune response upon infection with *A. hydrophila* [7]. However, it was not verified whether the feeds lowered the bacterial population in the fish. This study aimed to bridge that gap.

## Methods

**Fig. 1.**
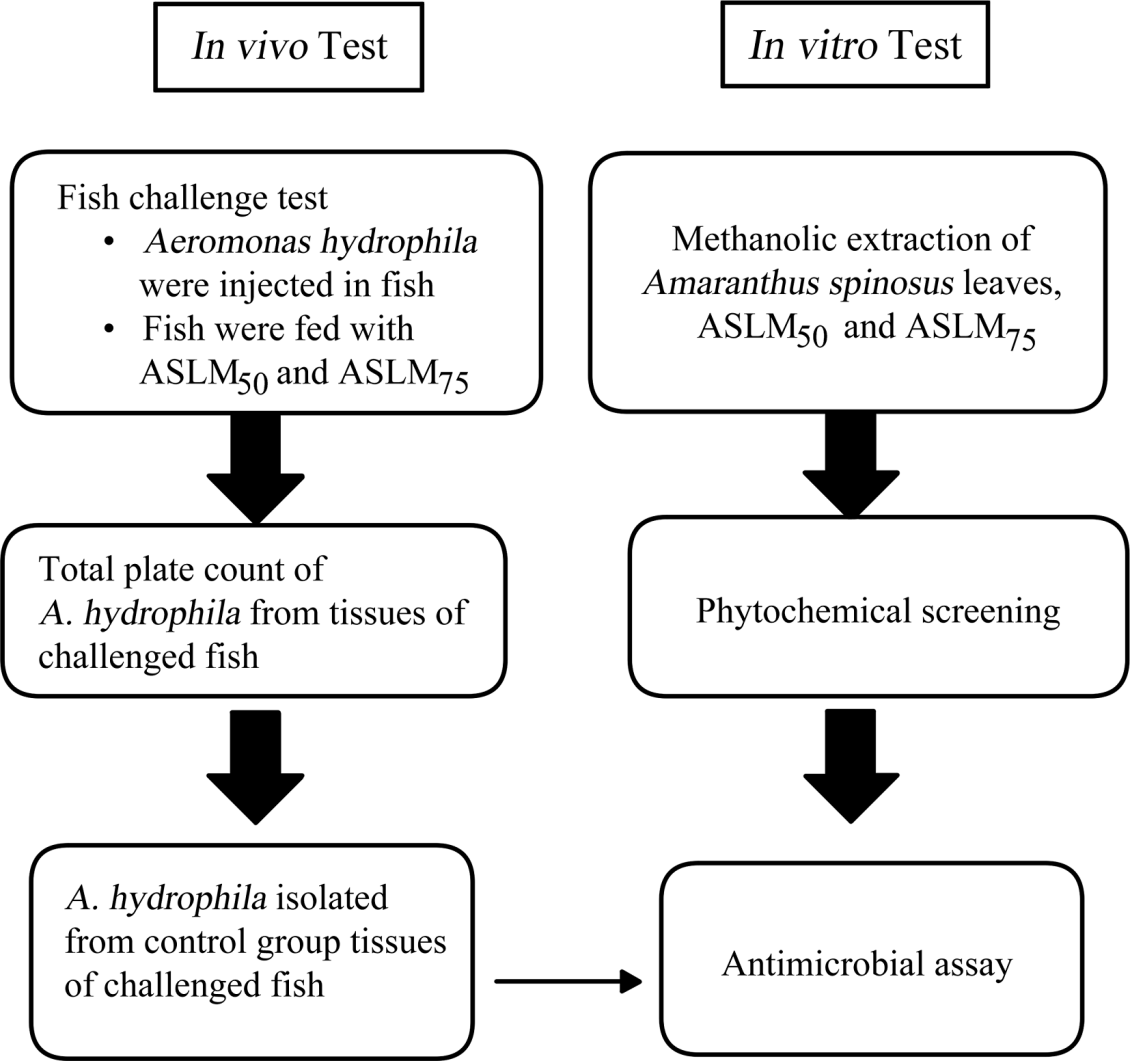
Diagram of the procedures performed to determine whether tilapia feeds with *Amaranthus spinosus* leaves (ASLMs) would be able to make Nile tilapia more resistant against *Aeromonas hydrophila* infections and if the leaves had a direct microbial effect on the pathogen.

The methodology of the study comprised *in vivo* and *in vitro* tests. A schematic diagram for the methods is presented in Fig. 1.

### Feed formulation

The study was conducted from January to December 2020. Leaves of *A. spinosus* were collected from cornfields in Echague, Isabela, Philippines. Preparation of *A. spinosus* L. leaf meals (ASLMs) was done at Isabela State University-Echague, Isabela. Feeds with 50 and 75% fish meal substitution with *A. spinosus* L. leaves, ASLM_50_ and ASLM_75_, respectively, were formulated [7]. Fish feed with 0 % *A. spinosus* leaves were also formulated and used as a control. The control [or basal feeds (BFs)] refers to standard tilapia feed formula without additives [7].

### Fish challenge test

Juvenile (50–60 g) male Nile tilapia (*Oreochromis niloticus*) were acquired from the Bureau of Fisheries and Aquatic Resources Region – VII (BFAR-VII), Bohol, Philippines. They were delivered and stored in a pool at the University of San Carlos – Talamban, Cebu, Philippines. The fish were acclimatized and fed with control feeds before experimentation. To reduce environmental interference, water parameters (dissolved oxygen, ammonia, pH, temperature) were monitored daily. In the study, average water temperature, pH, dissolved oxygen and ammonia were 26.07 °C, 8.04, 5.02 mg l^−1^ and 0.01 p.p.m., respectively. Whenever parameters went over the optimum [8], the water was changed or adjusted with sterile freshwater. The water was changed every 3 days and the photoperiod was maintained at 12 h light and 12 h darkness.

After a week of acclimatization, fish were distributed to 120 l plastic tubs in a completely randomized design. Each individual was inspected to be disease free. The screening was based on the external morphological signs of infected fish (e.g. bloated stomach, sluggish swimming behaviour, fin rot) [3]. A licensed fish pathologist took charge of the assessment. The experiment had four trials; each tub served as a trial. A total of 12 tubs (4 trials per treatment) with 10 individuals were set up. Fish were fed with 10 % of the bulk weight in each tub. Feeding was done twice daily (1000 and 1600 h). Fish were given formulated feeds corresponding to their designated treatment [ASLM_50_, ASLM_75_, or basal feeds (BF)/control).

After 2 weeks of feeding, a challenge test followed. Fish were starved for 12 h and then intraperitoneally injected with 0.2 ml of approximately 10^7^ colony-forming units (c.f.u.) ml^−1^ *A. hydrophila* (BIOTECH 10089) suspended in normal saline solution (NSS) [7]. Tricaine methanesulfonate (MS 222) was used as anaesthetic. Before returning the fish to the tank, it was ensured that they recovered from the anaesthetic.

After challenge with *A. hydrophila *, fish were monitored for 10 days. They were fed continuously with their designated treatments. Signs of disease were observed. Checking of environmental parameters and water change was still carried out. After the 10th day, all the fish were euthanized by prolonged submersion in MS 222.

### Total bacterial count from challenged fish

After the challenge test, 9 randomly selected fish per treatment were dissected (*n*=36). The spleen and kidney of each fish were removed. The organs were placed in preweighed microcentrifuge tubes with 500 µl of buffered peptone water (BPW) to rinse collected tissues [9]. Based on the organ’s fresh weight, new buffered peptone water was added to have 1 : 10 ratio (w/v) tissue suspension. The organs were then homogenized with a micropestle and contents were mixed using a vortex to achieved an initial dilution of ~10^−1^. Then the initial dilution was serially diluted up to a factor of 10^−3^. Twenty microlitres from each dilution was pipetted onto plates with corresponding labels. These were spread plated on the selective medium, Inositol Brilliant Green Bile Agar (HiMedia M574) and nutrient agar. Then the plates were incubated for 24 h at 35 °C and the total number of colonies from each organ was counted and expressed as c.f.u. g^−1^. The selective medium (IBGBA) only allowed the growth of the enteric bacteria *Plesiomonas shigelloides *, *Klebsiella* and *Aeromonas* [10]. The colonies of *Aeromonas* were distinguished from the other two genera based on colony colour and appearance on the selective medium [10].

### Extraction of *A. spinosus* L. leaves, ASLM_50_ and ASLM_75_

Before extraction, *A. spinosus* plant from Isabela State University, Echague City, Isabela was authenticated in the University of San Carlos Biology Department by presenting leaf, flower and fruit samples acquired from the place.

### Collection and preparation of *A. spinosus* L. leaves

Fresh *A. spinosus* L. leaves were acquired from different areas in Echague City, Isabela province (composite sampling). The plant was abundant in the cornfields and riversides. The leaves were air-dried and ground into powder by a pulverizing machine.

### Crude methanolic extraction of *A. spinosus* L. leaves and leaf meals

*A. spinosus* L. leaf (ASL), leaf meal (ASLMs) and basal feed (BF) powders were prepared for crude extraction. ASL refers to powders derived solely from the plant’s leaves, ASLM refers to the leaves at different concentrations (50 %, 75 %) plus other fish feed ingredients [7] and BF pertains to standard fish feed formula without any addition (control) [7].

Powders were extracted using methanol as the solvent that can draw out the most phytochemicals [11] .The methods for plant extraction were based on the study of Bulbul *et al.* (2011) [12] with slight modifications. ASL, ASLM_50_, ASLM_75_ and BF (120 g) powders were extracted exhaustively with a soxhlet apparatus for 9 h with methanol. The temperature was set to 60 °C [13]. Exhaustive extraction was attained when methanol in the soxhlet apparatus produced a clear solution. Organic material collected after total evaporation of methanol through rotary evaporation was stored in a scintillation vial.

### Phytochemical screening

The methanolic extracts of the ASL, ASLM_50_, ASLM_75_ and BF were qualitatively checked for the presence of phytochemical components. The procedures for phytochemical screening were based on Sable and Saswade (2017) [14], as outlined in their paper, and were performed in triplicate. The phytochemical constituents tested were alkaloids, carbohydrates, cardiac glycosides, flavonoids, phenols, amino acids and proteins, saponins, tannins, terpenoids, quinones and coumarins.

### Qualitative testing of crude methanolic extracts (ASL, ASLM) for direct microbial activity against *A. hydrophila*

#### Test organism and growth conditions

*A. hydrophila* recovered from the plated tissues of control group fish from the challenge test were isolated and cultured in nutrient agar at 28 °C. This was the main test organism used for the determination of antimicrobial activity.

### Antimicrobial activity of ASL and ASLM extracts against recovered *A. hydrophila* – resazurin-based broth microdilution method

An antimicrobial assay was performed to check the microbial activity of the extracts against the pathogen. The minimum inhibitory concentration (MIC) and minimum bactericidal concentration (MBC) of the extracts were determined using the resazurin-based microdilution method.

Prior to initiating the assays, the concentrated ASL, ASLM_50_, ASLM_75_ and BF methanolic extracts were weighed and dissolved in dimethyl sulfoxide (DMSO) (Scharlau). Agar well diffusion was first performed to determine the range of possible extract concentrations with bioactivity (500, 250, 125 mg, 50 mg and 10 mg ml^−1^). The negative controls were methanol and DMSO, whereas the positive control was oxytetracycline (2 mg ml^−1^). A 24 h culture of *A. hydrophila* recovered from the challenge test was used as the test organism. The lowest concentration of the extracts that showed clear zones was used for the microdilution method.

### Microplate preparation

**Fig. 2.**
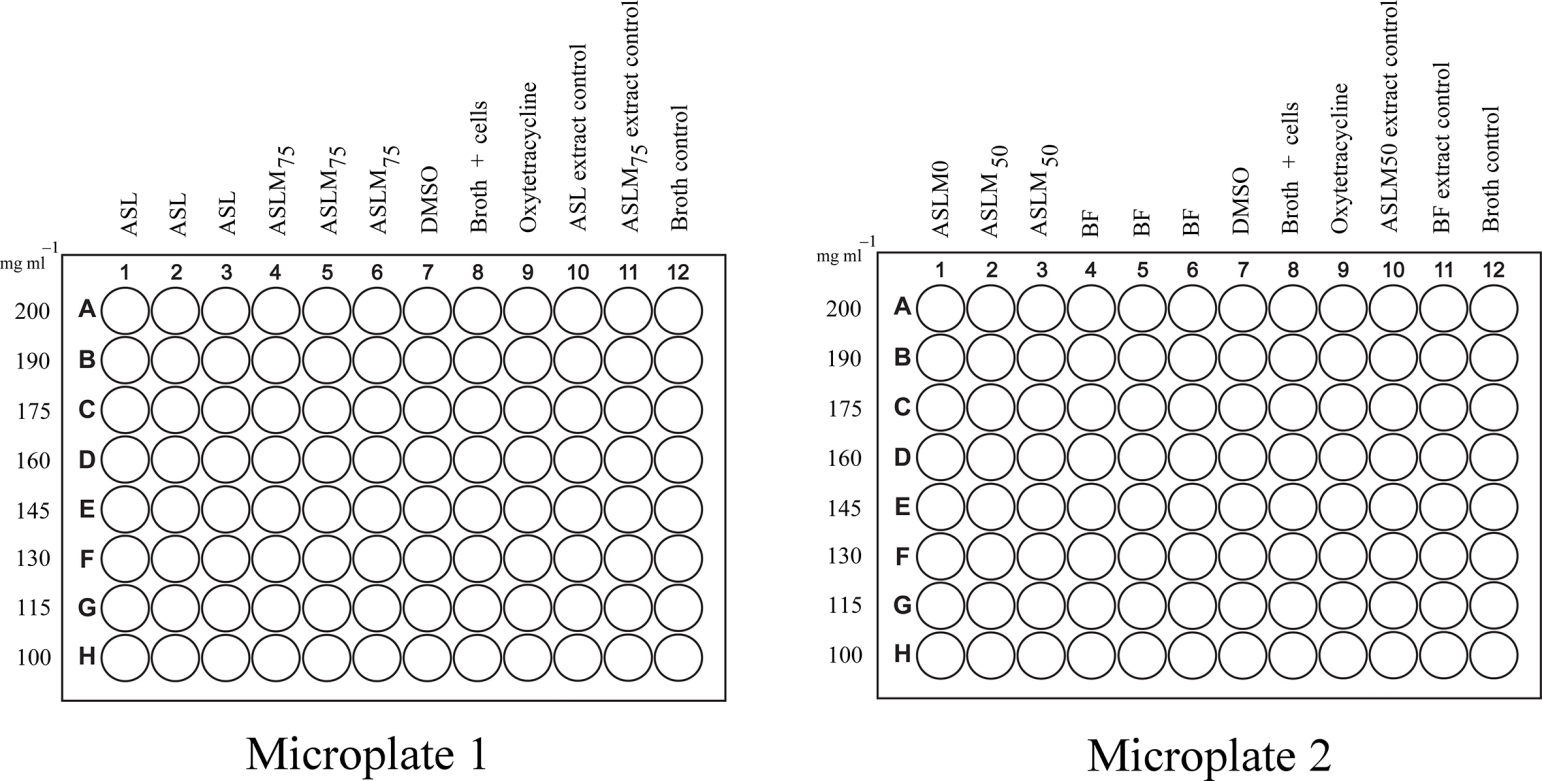
Microplate assay set-up for the resazurin-based microdilution method. Crude methanolic extracts were tested at different concentrations. Each plate represented one trial with three replicates per extract. The test bacteria used were only *A. hydrophila* recovered from the Nile tilapia challenge test. ASL, *A. spinosus* leaf extract; ASLM_75_, *A. spinosus* leaf meal with 75 % *A. spinosus* leaves used to substitute fish meals in standard feed formulation [7]; ASLM_50_ *, A. spinosus* leaf meal with 50 % *A. spinosus* leaves used to substitute fish meals in standard feed formulation [7]; BF, basal feed or control feed, standard feed formulation with no addition of *A. spinosus* leaves.

Broth microdilution assay was performed using a flat bottom 96-well microplate. Concentrations that ranged from 400 to 200 mg ml^−1^ with increments of 15 were prepared for the ASL, ASLM_50_, ASLM_75_ and BF methanolic extracts. With the use of a multichannel micropipette, 50 μl of Mueller–Hinton broth (MHB) (HiMedia M391) was added to the wells designated for the extracts (columns 1–6), negative control (column 7) and positive control (column 9). The positive control was oxytetracycline (2 mg ml^−1^) (Sigma-Aldrich) and the negative control was 10 % DMSO aqueous solution. Fifty microlitres of the prepared extract concentrations was added to the designated wells. Wells in row A had the highest concentration and the concentrations were successively decreased. Row H had the lowest concentration of added extracts. This diluted the initial concentration of the extracts by half. Wells in columns 8, 10 and 11 had 100 μl broth added. Columns 10 and 11 served as the extract control. Column 12 had 200 μl of MHB added. This column served as the media sterility control. Fig. 2 shows the microplate set-up.

### Addition of test organism in microplate

A spectrophotometer [PASCO Wireless Spectrometer (VIS) • PS-2600] was used to determine the turbidity of the inoculum at 600 nm. The inoculum was adjusted until it was comparable to that of a 0.5 McFarland standard (0.063). Serial dilution was then performed to reach a final concentration of 10^5^ c.f.u. ml^−1^ inoculum. A multichannel micropipette was used to load 100 µl of the bacteria into the wells of columns 1–9. The final volume of all the wells in each microplate was 200 µl. Microplates were then incubated for 24 h at 28 °C.

### Resazurin assay

After incubation, a resazurin assay was conducted. In a dark room, a multichannel micropipette was used to add 15 µl of 0.015 % resazurin dye (Sigma-Aldrich) to all the wells. The microplate was then incubated in the dark. The colour of wells after 24 h of dye addition was observed. A colour change from blue to pink signified the presence of metabolically active bacterial cells in the well. The dye indicates metabolic activity because its colour changes depending on a redox reaction caused by active cell metabolism [15]. Colour changes were noted.

### Spot test

A spot test was performed after the resazurin assay. This was done to verify the results of the resazurin test and determine the MBC. Pipette tips were dipped in the wells and gently touched on nutrient agar plates that had corresponding labels. Plates were incubated at 28 °C for 24 h and bacterial growth was observed. The concentration where no bacterial growth was observed was assigned as the MBC. Concentrations where bacterial spots grew but had a blue colour in the assay were designated as the MIC.

### Statistical analysis

One-way analysis of variance (ANOVA) was used to verify whether the amount of *A. spinosus* in the leaf meals was able to affect the counts of *A. hydrophila* found in infected Nile tilapia.

The colony counts were log-transformed to normalize the data before ANOVA. Tukey’s test for post-hoc analysis was used to pinpoint which groups were different when *P*-values were significantly different (*P*<0.05). The level of significance applied for all analysis was at 95 % confidence. Minitab17 software (trial version) (Pennsylvania, USA) was used for all calculations.

## Results

### Total bacterial count from challenged fish

No mortalities occurred during the challenge test. External and internal signs of *Aeromonas* infections were evident in all the fish even in the control group. Haemorrhage at the base of the fins and inflammation of internal organs (liver, spleen and kidneys) were observed in all the groups. Based on visual observation, it was noted that fish from the control group had the most inflamed organs, especially the liver. All visual observations on the pathogenesis of the bacteria were assessed and verified by a licensed fish pathologist.

**Fig. 3.**
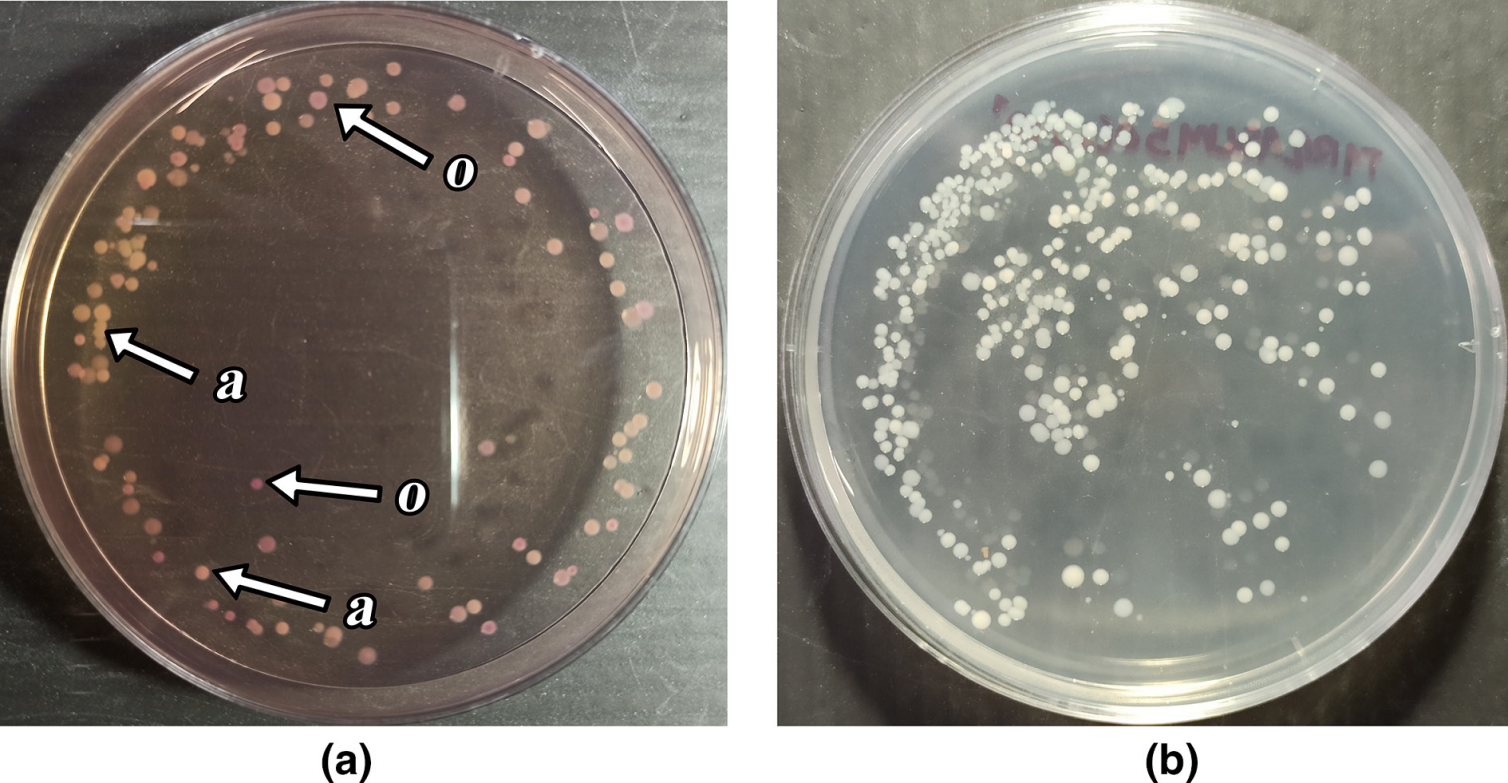
Bacterial colony appearance in media (Himedia 2011). (**a**) Colourless *Aeromonas hydrophila* colonies in Inositol Brilliant Green Bile Agar. (**b**) Bacteria in nutrient agar. a, *Aeromonas *; o, other bacteria (* Klebsiella pneumoniae* or *Plesiomonas shigelloides *).

The results showed a significant difference between the counts of enteric bacteria (*P*=0.019) and *A. hydrophila* (*P*=0.03) only occurred in the spleen, but no significant differences in the number of bacteria were detected from the kidneys (Tables 1 and 2). Representative photographs of how the bacteria appeared on the media used are shown in Fig. 3.

**Table 1.** Colony-forming units (c.f.u.) of bacteria recovered from challenged Nile tilapia kidneys at different treatments (*n*=36)

Treatment	Wet kidney WT (g)	SD	Total bacterial count (c.f.u. g^−1^)	SD	Enteric bacteria (c.f.u. g^−1^)	SD	* Aeromonas * (c.f.u. g^−1^)	SD
Control	0.0523	±0.061	3.79×10^4^	±5.58×10^4^	7.78×10^4^	±1.68×10^5^	5.70×10^4^	±1.24×10^5^
ASLM50	0.0461	±0.050	5.56×10^4^	±1.81×10^6^	4.46×10^4^	±1.00×10^5^	2.73×10^4^	±5.83×10^4^
ASLM75	0.0417	±0.041	6.45×10^4^	±1.60×10^5^	1.32×10^5^	±2.85×10^5^	5.72×10^4^	±1.19×10^5^
			*P=0.824*		*P=0.707*		*P=0.648*	

**Table 2.** Colony forming units (c.f.u.) of bacteria recovered from challenged Nile tilapia spleen at different treatments (*n*=36)

Treatment	Wet spleen WT (g)	sd	Total bacterial count (c.f.u. g^−1^)	sd	Enteric bacteria (c.f.u. g^−1^)	sd	* Aeromonas * (c.f.u. g^−1^)	sd
Control	0.2234	±0.110	1.3×10^5^	±2.26×10^5^	6.13×10^4^	±1.35×10^5^	5.71×10^4^	±1.34×10^5^
ASLM50	0.1999	±0.080	4.93×10^4^	±1.49×10^5^	2.92×10^3^	±4.4×10^3^	1.75×10^3^	±2.78×10^3^
ASLM75	0.2078	±0.072	5.99×10^5^	±1.72×10^6^	3.75×10^5^	±8.59×10^5^	2.82×10^5^	±6.52×10^5^
			*P=0.89*		*P=0.019*		*P=0.03*	

Tukey’s post-hoc test revealed that the number of enteric bacteria from fish fed with ASLM_50_ was significantly different from those that were fed with ASLM_75_ and control feeds. Counts from fish fed with ASLM_50_ were significantly different in the spleen from those that were fed with ASLM_75_ but not with those fed with control feeds.

### Phytochemical screening

Phytochemical screening for the crude methanolic extracts of the ASL, ASLM_50_, ASLM_75_ and BF of this study are shown in Table 3. The compounds present in the crude methanolic extracts were carbohydrates, cardiac glycosides, amino acids, proteins and terpenoids. Saponins and steroids were only detected in the extracts of ASL and ASLM_75_.

**Table 3.** Phytochemical screening of crude methanolic extracts of ASL, ASLM_75_, ASLM_50_ and BF.

Phytochemical constituent	Method	Positive result	*A.* leaves (ASLs)	ASLM_75_	ASLM_50_	Basal feeds (BFs)
Alkaloids	Wagner’s test	Reddish brown coloration	−	−	−	−
Carbohydrates	Molisch’s test	Purple ring at the interface	**+**	**+**	**+**	**+**
Cardiac glycosides	Keller Kelliani test	Brown ring at the interface	**+**	**+**	**+**	**+**
Flavonoids	Lead Acetate test	Disappearance of yellow coloration	−	−	−	−
Phenols	Ferric Chloride test	Deep blue or black coloration	−	−	−	−
Amino acid and protein	1 % ninhydrin in acetone	Purple colour	**+**	**+**	**+**	**+**
Saponins	Foam test	Foam layer	**+**	**+**	−	−
Tannins	Braymers test	Blue/greenish colour	−	−	−	−
Terpenoids	Salkowski test	Yellow coloration	**+**	**+**	**+**	**+**
Steroids	Liebermann–Burchard’s test	Colour change from violet to blue or green	**+**	**+**	−	−
Coumarins	10 % NaOH	Yellow colour	−	−	−	−
Quinones	Sulfuric acid	Red colour	−	−	−	−

ASL, *A. spinosus* leaf extract; ASLM_75_ *A. spinosus* leaf meal with 75 % *A. spinosus* leaves used to substitute fish meals in standard feed formulation [7]; ASLM50, *A. spinosus* leaf meal with 50 % *A. spinosus* leaves used to substitute fish meals in standard feed formulation [7]; BF basal or control feeds, standard feed formulation with no addition of *A. spinosus* leaves.

### Qualitative testing of crude methanolic extracts (ASL, ASLM) for direct microbial activity against *A. hydrophila*

### Resazurin-based broth microdilution assay

The agar well diffusion range finding showed that the 300 mg ml^−1^ was the lowest concentration where the extracts had bioactivity against the *A. hydrophila* recovered from the challenge test (Table 4). However, upon close observation of the plates, it was seen that at 200 mg ml^−1^, inhibitory zones formed, but these were later colonized by the bacteria. Based on this, the extracts were microdiluted to determine the possible MIC and MBC. Final concentrations of 200, 190, 175, 160, 145, 130, 115 and 100 mg ml^−1^ were prepared and used in the resazurin-based broth microdilution assay.

**Table 4.** Agar well diffusion was used for range finding of extract minimum inhibitory concentration. (MIC) The zone of inhibition (ZOI) of the crude methanolic extracts against *Aeromonas hydrophila* recovered from Nile tilapia tissue are presented.

Zone of Inhibition (mm)*									
Extract concentration (mg ml^−1^)							(+) control	(−) control	(−) control
Sample	450	400	350	300	250	200	Oxytetracycline	Methanol	DMSO
ASL	17.40±1.45	15.38±1.60	13.20±1.16	9.60±2.19	5.50±0.93	5.00±0	35.53±0.93	5.00±0	5.00±0
ASLM75	12.25±1.10	8.99±0.78	6.23±1.33	5.00±0	5.00±0	5.00±0			
ASLM50	16.76±1.41	12.28±1.36	7.44±1.64	5.75±1.39	5.00±0	5.00±0			
BF	5.90±1.57	5.00±0	5.00±0	5.00±0	5.00±0	5.00±0			

*Diameter of wells is 5 mm; 5 mm, no inhibition.

ASL, *A. spinosus* leaf extract; ASLM75 *A. spinosus* leaf meal with 75% *A. spinosus* leaves used to substitute fishmeals in standard feed formulation [7]; ASLM50, *A. spinosus* leaf meal with 50 % *A. spinosus* leaves used to substitute fishmeals in standard feed formulation [7]; BF basal or control feeds, standard feed formulation with no addition of *A. spinosus* leaves.

**Fig. 4.**
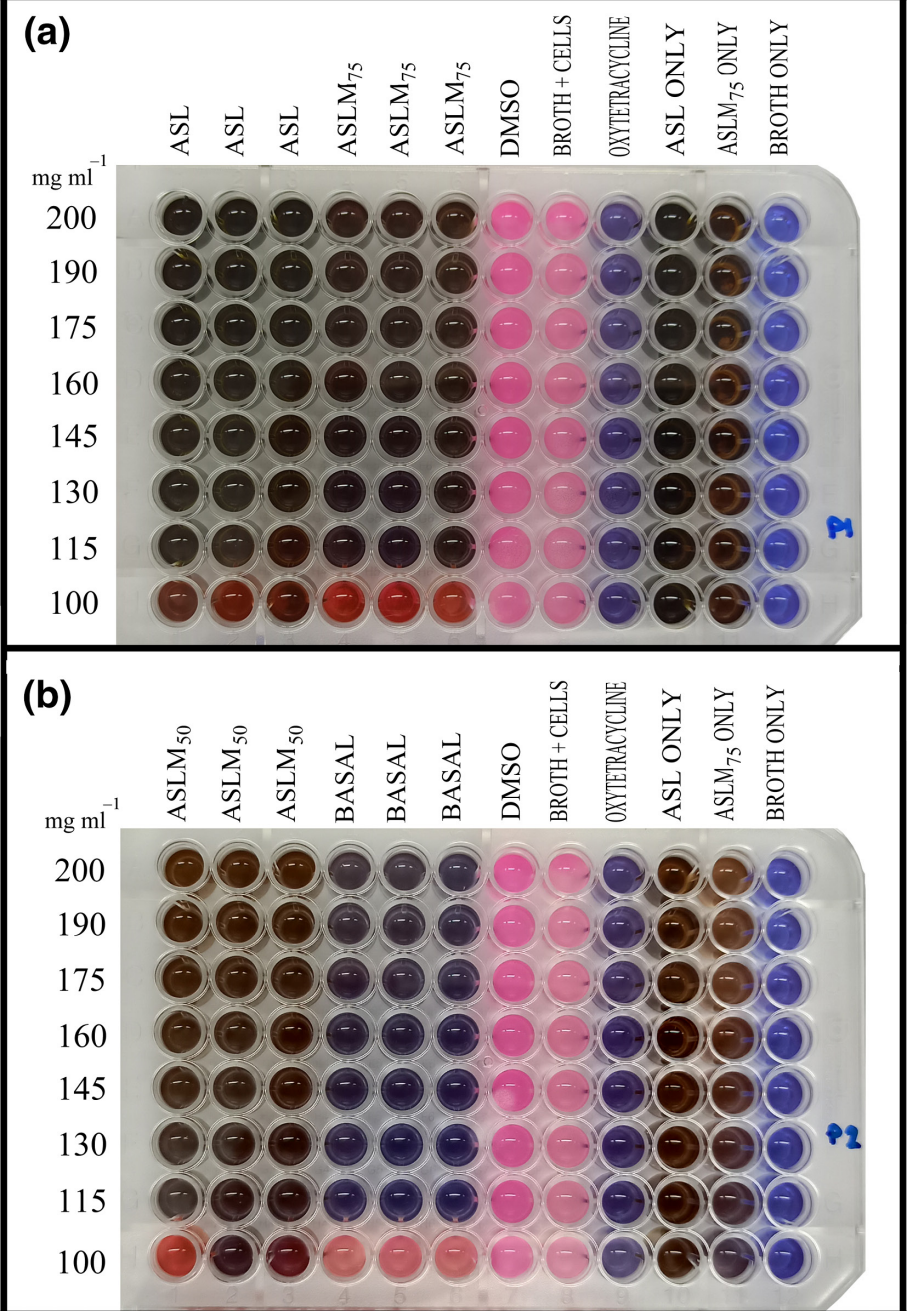
Microplate 24 h after addition of resazurin. (**a**) Microplate containing ASL and ASLM_75_ extract replicates. (**b**) Microplate containing ASLM_50_ and BF extract replicates. ASL, *A. spinosus* leaf extract; ASLM_75_, *A. spinosus* leaf meal with 75 % *A. spinosus* leaves used to substitute fish meals in standard feed formulation [7]; ASLM_50_, *A. spinosus* leaf meal with 50 % *A. spinosus* leaves used to substitute fish meals in standard feed formulation [7]; BF, basal feeds or control feeds, standard feed formulation with no addition of *A. spinosus* leaves.

Fig. 4 depicts the microplates after the resazurin assay was performed. The ASL, ASLM_75_, ASLM_50_ and BF extracts had a colour change at 100 mg ml^−1^ but at 115–200 mg ml^−1^, there was no change. The negative controls (DMSO and MHB) had a colour change, implying that the solvent of the extracts (DMSO) had no effect on the bacteria and that the media being used (MHB) allowed bacterial growth. No colour change occurred in any wells of the positive control, oxytetracycline (2 mg ml^−1^) and in the sterility controls (broth and extracts alone). These results signified that the bacteria were susceptible to the positive control and that the broth and extracts being used were all sterile.

### Spot test

**Fig. 5.**
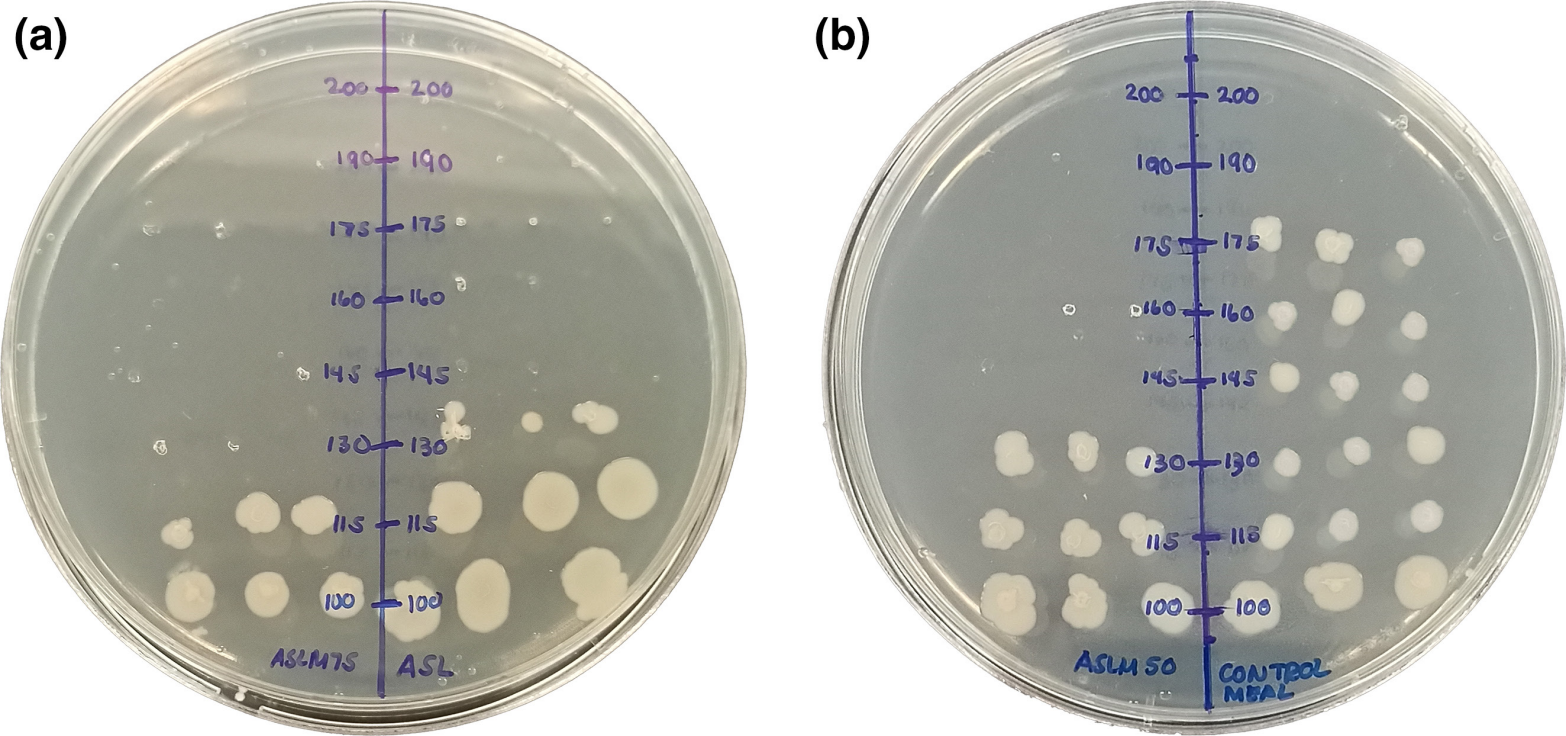
Spot test of microplate contents. (**a**) Plate for spot tests of ASL and ASLM_75_ extract concentrations. (**b**) Plate for spot tests of ASLM_50_ and basal or control feeds extract concentrations. ASL, *A. spinosus* leaf extract; ASLM_75_, *A. spinosus* leaf meal with 75 % *A. spinosus* leaves used to substitute fish meals in standard feed formulation [7]; ASLM_50_, *A. spinosus* leaf meal with 50 % *A. spinosus* leaves used to substitute fish meals in standard feed formulation [7]; BF, basal feeds or control feeds, standard feed formulation with no addition of *A. spinosus* leaves.

Fig. 5 shows the results of the spot test. The concentration where no bacteria grew was considered to be the MBC value. The extract with the lowest MBC (most potent) was the ASLM_75_, as bacteria only started growing at 115 mg ml^−1^, whereas the control feeds or BF had the highest MBC (least bactericidal), since at 175 mg ml^−1^, bacteria had already started growing. Table 5 summarizes the MIC and MBC of the crude methanolic extracts, as evidence for the direct microbial activity of the leaves against the pathogen.

**Table 5.** Minimum inhibitory concentration (MIC) and minimum bactericidal concentration (MBC) values of extracts against *Aeromonas hydrophila* recovered from Nile tilapia tissue.

	MIC (mg ml^−1^)	MBC (mg ml^−1^)
ASL	115	145
ASLM75	115	130
ASLM50	115	145
BF	115	190

ASL, *A. spinosus* leaf extract; ASLM75, *A. spinosus* leaf meal with 75 % *A. spinosus* leaves used to substitute fishmeals in standard feed formulation [7]; ASLM50, *A. spinosus* leaf meal with 50 % *A. spinosus* leaves used to substitute fishmeals in standard feed formulation [7]; BF, basal feeds or control feeds, standard feed formulation with no addition of *A. spinosus* leaves.

## Discussion

Most challenge tests are conducted under a longer duration, especially with *A. hydrophila *, since this pathogen is not very virulent [16]. However, these challenge tests are conducted for survivability or growth studies, whereas this study showed how the pathogen *A. hydrophila* was affected by the experimental fed diet (ASLM) of its host.

Inflammation of internal organs is common among the fish affected by *Aeromonas* species [3, 17]. Inflammation occurs because the affected organs such as the spleen and kidney work laboriously to compensate for the damage done by the bacterial infection [18]. This affirmed that the inoculum of *A. hydrophila* during the challenge test was virulent and caused fish systemic infection.

The spleen and kidneys of fish are some of the first organs affected by *A. hydrophila* during infection. The pathogen is known to attack blood cells and causes haemolysis due to its surface structures, enterotoxins and extracellular products [19]. The analyses showed that ASLM_50_ significantly lowered the number of enteric bacteria and *A. hydrophila* in the spleen of Nile tilapia but not in the kidneys. The kidneys may not have been the best organ for recovering bacteria in this type of study, although the isolation of kidneys was performed uniformly in all the treatments and trials to prevent bias.

This could be attributed to the difficulty in isolating the entire fragile organ. In this study, most of the kidneys isolated were derived from the middle section (directly above digestive system) and less often from the anterior sections (near the eyes). The numbers of bacteria recovered from the fish kidneys may have been different if the entire organs were isolated. This is because the sections in the kidneys of fish have various cells that perform different functions depending on their position. The anterior section is more concerned with haematopoiesis, while the rest of the organ performs immune functions [20]. *A. hydrophila* is a pathogen that attacks blood cells.

The bacterial counts from fish fed with ASLM_50_ were lower than those for those fed with the control and ASLM_75_. This finding is similar to those from other studies that have tried to determine the performance of fish or other aquatic organisms fed with plant-infused diets and then challenged with a pathogen [21, 22]. They are similar in the sense that a higher substitution of plant-based protein (PP) does not necessarily yield better performance in the organism. It was shown that freshwater-cultured shrimps (*Macrobrachium nipponense*) had better growth and resistance against *A. hydrophila* when their fish meals (FMs) were replaced with 25 % soybean meal [21]. An increased percentage of PP of >25 % resulted in shrimps with lower growth performance and less bacterial resistance than those fed with pure FMs. An investigation of the effects of substituting FMs with different sources of PPs (corn, wheat, rapeseed and white lupin) towards the non-specific defence mechanisms and oxidative stress of gilthead sea bream (*Sparus aurata*) was also performed [22]. It was reported that fishes fed with any of the PPs at 100 and 75% substitution of FMs had compromised growth and lowered immune defence. PPs usually have the best results on the growth and performance of fish or other farmed aquatic animals when they substitute fish meals within 25–50 % [21, 22]. In this study, the 50 % substitution of FMs with *A. spinosus* leaves had better results than pure FMs and feeds with 75 % *A. spinosus*. These results were also similar to the study of Angeles *et al.* (2020) [7] where challenged Nile tilapia fed with ASLM_50_ had better growth performance compared to those fed with ASLM_75_ and control feeds. In their study, tilapia fed with ASLM_50_ had more weight gain than those fed with ASLM_75_ and control/basal feeds.

A possible explanation for why high levels of PPs integrated into fish feed does not necessarily lead to better fish performance is the presence of antinutritional factors (ANFs). ANFs are defined as normal metabolic byproducts that compromise optimal nutrition and are derived after the consumption of naturally occurring compounds found in foodstuffs [23]. Secondary metabolites in plants (i.e. saponins, tannins and flavonoids) can potentially become ANFs. Being an ANF is not an intrinsic property of a compound; rather it is dependent on the digestion or metabolism of the animal consuming it [23]. Plant secondary metabolites can have beneficial or harmful effects on an animal, depending on their amount and the consumer’s metabolism. The physiological effects of the metabolites found in a plant on its intended consumer have to be determined so that the appropriate level of plant or metabolite inclusion can be quantitatively known. Therefore, plant-derived products must be properly processed and studied before being sold commercially [24].

In this study, colony counts were used in determining the effect of the feeds on bacteria when inside the fish. Most studies of this kind use haematological profiles, mortalities and growth of the fish to evaluate the effect of their experimental diets when the fish are challenged. From there, they also assume that the pathogen is affected. However, these methods do not show the effect of the feeds on the pathogen, but focus more on the fish. A commonly known fish pathogen such as *A. hydrophila* may just be opportunistic and not necessarily be the cause of fish mortality or leukocyte increase. Microbes in the natural environment always exist in communities [25]. Their interactions can lead to the presence of metabolites that can be either good for or harmful to the host. Hence, total bacterial counts on nutrient agar (Tables 1 and 2) were also noted. In both organs, the counts of general bacteria were not significantly different among the fish fed with different diets and they were higher than the counts of *A. hydrophila *. This means that other bacteria apart from *A. hydrophila* were also present in the organs and were possibly not affected by the changes in feed formulation. However, taxonomic identification of the bacteria recovered in nutrient agar was not traced in the study.

In the Philippines, Pakingking *et al.* (2015) [26] investigated the taxonomic profile of microbiota recovered from the gills and intestines of tilapia that were cultured in earthern ponds and were not necessarily diseased. Their study showed that most bacteria detected are opportunistic pathogens such as *Aeromonas* spp., *Pasteurella* spp., *Plesiomonas shigelloides* and *Vibrio* spp. The bacteria with the highest recovery were *A. hydrophila* (31.4 %). This shows that opportunistic pathogens such as *A. hydrophila* can become problematic, especially when they reach cell densities that can elicit disease, as occurred during the challenge test of this study. Hence, actions should be taken against the bacteria before they emerge as a dire problem.

The findings of the challenge test (*in vivo*) indicated that *A. spinsosus* leaf meals (ASLMs) inhibited the pathogen inside the fish. However, the *in vivo* results did not necessarily prove this because other factors might have contributed to the effect. The ASLMs might have boosted the fish immune system as *A. spinosus* has been reported to be a great source of antioxidants such as amaranthine [11]. Antioxidants help increase fish immune system parameters (e.g. immunoglobulins, interleukins) that enhance their resilience against bacteria [27]. Angeles *et al.* (2019) reported the ability of ASLMs to boost the immune system of *A. hydrophila* challenged Nile tilapia [7]; however, possible direct effect of the ASLMs on the bacteria was not investigated.

The phytochemical screening showed that the plant leaves contained carbohydrates, amino acids and protein, cardiac glycosides, saponins and terpenoids (Table 3). Cardiac glycosides and saponins are generally classified as glycosides. These two glycosides have a steroid attached to a sugar molecule [28]. This explains why the broad group of phytochemicals such as steroids and carbohydrates were detected (Table 3).

Cardiac glycosides are composed of an aglycone (cardenolide or bufadienolide) attached to a sugar molecule [29] and are known for their inhibitory effects on sodium potassium pumps of cell membranes, especially those of cardiac cells. This contributes to their prominent use as anti-arrhythmic agents. A study pointed out that cardiac glycosides alone dos not have antimicrobial effects, but can inhibit bacterial growth in the presence of other phytochemical compounds [30].

The antimicrobial activity of saponins and terpenoids is well documented [31]. Saponins are diverse metabolites composed of an aglycone (triterpenoid or steroidal) attached to a sugar chain. Terpenoids are also diverse metabolites with isopentenyl diphosphate units as a backbone. Bioactive compounds (e.g. lycopene, astaxanthin, gibberellic acids) with various activities are derived from its backbone [32]. Terpenoid derivatives inhibit the cell walls of bacteria, contributing to their antimicrobial effect [33].

Koch’s postulates convey that when a pathogen is inoculated into a healthy individual, the individual will exhibit the symptoms associated with that pathogen and the pathogen derived from the diseased invidual is closer to its virulent state than when outside the body. In this study, the bacteria used in the microbial activity assays were *A. hydrophila* recovered from the challenged Nile tilapia (*O. niloticus)*. The study showed that the crude methanolic extracts of *A. spinosus* leaves and meals exhibited antimicrobial activity (Table 5). This implies that the feeds not only boosted the immune system of the fish [7], but they also had bioactive compounds that were able to affect the pathogen. Between ASLM_75_ and ASLM_50_, ASLM_50_ had the better effect on the fish’s resilience against the bacteria (Tables 1 and 2). This was discussed as being an effect of antinutritional factors. The phytochemical screening corroborates with this, as cardiac glycosides when found in higher concentrations are reported to have physiologically negative effects [29].

In summary, the study showed the promising potential of *A. spinosus* leaves to be used as a substitute for fish meals because, aside from its high-quality protein, the plant itself is an agricultural pest – utilizing it does not compete with human consumption, making it a sustainable source. Upon consumption of feeds with *A. spinosus* leaf meal (ASLM), Nile tilapia can also become more resilient against *A. hydrophila* infections, as seen by the lower recovery of bacterial cells (c.f.u. g^−1^) from its tissues after infection. Direct antimicrobial effects of *A. spinosus* extracts on the pathogen were also reported. These findings can help Nile tilapia farmers cut down on expenses as ASLM is cheaper to produce and it also lessens the need for antibiotics.
